# Self-harm in primary school-aged children: Prospective cohort study

**DOI:** 10.1371/journal.pone.0242802

**Published:** 2020-11-30

**Authors:** Rohan Borschmann, Lisa K. Mundy, Louise Canterford, Margarita Moreno-Betancur, Paul A. Moran, Nicholas B. Allen, Russell M. Viner, Louisa Degenhardt, Silja Kosola, Izabela Fedyszyn, George C. Patton

**Affiliations:** 1 Justice Health Unit, Centre for Health Equity, Melbourne School of Population and Global Health, The University of Melbourne, Melbourne, Victoria, Australia; 2 Centre for Adolescent Health, Murdoch Children’s Research Institute, Melbourne, Victoria, Australia; 3 Health Service and Population Research Department, Institute of Psychiatry, Psychology & Neuroscience, King’s College London, London, United Kingdom; 4 Melbourne School of Psychological Sciences, The University of Melbourne, Melbourne, Victoria, Australia; 5 Department of Paediatrics, The University of Melbourne, Melbourne, Victoria, Australia; 6 Clinical Epidemiology and Biostatistics Unit, Murdoch Children’s Research Institute, Melbourne, Victoria, Australia; 7 Centre for Epidemiology and Biostatistics, Melbourne School of Global and Population Health, The University of Melbourne, Melbourne, Victoria, Australia; 8 Centre for Academic Mental Health, Department of Population Health Sciences, Bristol Medical School, University of Bristol, Bristol, United Kingdom; 9 Department of Psychology, University of Oregon, Eugene, Oregon, United States of America; 10 Children’s Policy Research Unit, University College London, London, United Kingdom; 11 National Drug and Alcohol Research Centre, UNSW Sydney, Sydney, NSW, Australia; 12 Pediatric Research Center, Helsinki Children’s Hospital, Helsinki University Hospital and University of Helsinki, Helsinki, Finland; Karolinska Institutet, SWEDEN

## Abstract

**Introduction:**

No prospective studies have examined the prevalence, antecedents or concurrent characteristics associated with self-harm in non-treatment-seeking primary school-aged children.

**Methods:**

In this cohort study from Melbourne, Australia we assessed 1239 children annually from age 8–9 years (wave 1) to 11–12 years (wave 4) on a range of health, social, educational and family measures. Past-year self-harm was assessed at wave 4. We estimated the prevalence of self-harm and used multivariable logistic regression to examine associations with concurrent and antecedent factors.

**Results:**

28 participants (3% of the 1059 with self-harm data; 18 girls [3%], 10 boys [2%]) reported self-harm at age 11–12 years. Antecedent (waves 1–3) predictors of self-harm were: persistent symptoms of depression (sex-age-socioeconomic status adjusted odds ratio [aOR]: 7.8; 95% confidence intervals [CI] 2.6 to 24) or anxiety (aOR: 5.1; 95%CI 2.1 to 12), frequent bullying victimisation (aOR: 24.6; 95%CI 3.8 to 158), and recent alcohol consumption (aOR: 2.9; 95%CI 1.2 to 7.1). Concurrent (wave 4) associations with self-harm were: having few friends (aOR: 8.7; 95%CI 3.2 to 24), poor emotional control (aOR: 4.2; 95%CI 1.9 to 9.6), antisocial behaviour (theft—aOR: 3.1; 95%CI 1.2 to 7.9; carrying a weapon—aOR: 6.9; 95%CI 3.1 to 15), and being in mid-puberty (aOR: 6.5; 95%CI 1.5 to 28) or late/post-puberty (aOR: 14.4; 95%CI 2.9 to 70).

**Conclusions:**

The focus of intervention efforts aimed at preventing and reducing adolescent self-harm should extend to primary school-aged children, with a focus on mental health and peer relationships during the pubertal transition.

## Introduction

Longitudinal and case-control studies have established that self-harm (defined as intentional self-poisoning or self-injury, irrespective of the motive or the extent of suicidal intent [1]) during adolescence is a risk factor for numerous adverse clinical and social outcomes [2–4], including suicide [5]. As such, the occurrence of self-harm in pre-teen children is a particularly concerning event [5, 6]. Yet to date, knowledge about self-harm in young people derives almost exclusively from adolescent populations [7–12], and few studies have examined self-harm in non-treatment-seeking young people aged 12 years or under [13–18]. This is a key period in the life course when the first symptoms of common mental disorders, such as depression and anxiety, begin to emerge [19, 20]. As such, no prevalence estimates of self-harm in primary school-aged children in the community have been published [1]. Much of what we know comes from treatment-seeking samples of adolescents [5, 10, 21–23], and the degree to which these findings are applicable to the general population remains unknown. Additionally, almost nothing is known about the characteristics of primary school-aged children who engage in self-harm, particularly in relation to pubertal stage [11]. This is important because pubertal stage has been linked to differing levels of risk for the onset of mental disorders and early substance abuse [19].

We addressed these notable gaps in the literature by analysing data from a prospective cohort study in Victoria, Australia: the Childhood to Adolescence Transition Study (CATS) [24]. We aimed to describe the cross-sectional correlates and antecedents of self-harm in primary school-aged children. In the absence of comparable data in the published literature on which to formulate specific a priori causal hypotheses, we conducted an exploratory analysis of CATS data to generate the first health, social and demographic profile of self-harm in this population.

## Methods

### Study population, design and procedure

Data for this study were drawn from CATS [24], a longitudinal cohort study with a broad focus on health, education and social adjustment as children make the transition from childhood to adolescence. Full details about the study design are reported elsewhere.[24] In brief, 2 289 students (aged 8–9 years) from a stratified random sample of 43 primary schools in Melbourne, Australia were invited to participate and 1 239 (54%) were recruited through informed parental consent. Participants were followed up annually from wave 1 (aged 8–9 years) to wave 4 (aged 11–12 years), with measures comprising student, parent and teacher questionnaires. Participation rates are presented in S1 Fig and further information about the measurement of depressive symptoms, anxiety symptoms, emotional control and overall wellbeing is located in the Supplementary Material under ‘Methodology’. Full details of the study design and protocol have been published elsewhere [24].

### Measures

#### Demographics

At wave 1, parents indicated their highest level of completed education and their child’s country of birth and Indigenous status. Socioeconomic status (SES) was calculated from each student’s home postcode using the 2011 Socio-Economic Index For Areas (SEIFA) Index of Relative Socio-economic Advantage and Disadvantage (IRSAD) [25]. Adjustment variables were age (measured at wave 4 student questionnaire completion, centred around 12.0 years) and sex.

#### Self-harm

At wave 4, participants were asked the following question: “In the past 12 months have you ever hurt yourself on purpose or done anything that might have harmed you or even killed you?” If participants responded “yes”, they were then asked to describe what they did. Free-text from this follow-up question was screened for self-harm using a coding system adapted from a recent large-scale epidemiological study of self-harm [9]. Self-harm was defined as any behaviour fitting into one of five categories: (1) cutting/burning, (2) self-poisoning, (3) self-battering, (4) non-recreational risk-taking (e.g., reckless behaviour near traffic), or (5) other self-harm.

#### Mental health and wellbeing

At each of waves 1–4, the presence of any depressive symptoms was measured using an adapted version of the Short Mood and Feelings Questionnaire (SMFQ) [26]. Anxiety symptoms were assessed using an adapted version of the Spence Children’s Anxiety Scale (SCAS) [27]. Emotional control was measured using four items from the International Youth Development Study (IYDS) [28–30]. Overall wellbeing was measured using the PedsQL General Wellbeing Scale [31].

#### Peer relationships

Participants were asked: “Do you have a group of friends?” with response options of ‘not many’, ‘some’, or ‘lots’. At waves 2–4, participants were also asked: “How often do you argue or fall out with your friends?” Peer victimisation was measured using selected items from the Gatehouse Bullying Scale [32] which assessed both overt (e.g., teasing) and covert (e.g., social exclusion) victimisation. An additional question on cyberbullying was included in waves 3 and 4.

***Pubertal development*** was assessed at wave 4 using the Pubertal Development Scale (PDS) [33]. An overall pubertal development score was created to categorise participants as either “pre-pubertal/early pubertal”, “mid-pubertal” or “late pubertal/post-pubertal” [33].

#### Academic performance and truancy

Teachers provided an overall rating of each participant’s abilities in English and mathematics using items adapted from the Longitudinal Study of Australian Children (LSAC) [34]. At wave 4, participants were asked whether they had deliberately skipped a lesson or left school without permission during the previous year.

#### Alcohol consumption

Participants were asked at wave 4: “Have you had more than a sip or taste of alcohol over the past 12 months?”

#### Antisocial behavior

Using items from IYDS [28–30], participants were asked whether they had carried a weapon, stolen something worth more than $5 or beaten someone so badly that the person required medical attention.

#### Family relationships

Items adapted from IYDS assessed whether participants felt they were able to discuss their feelings with their mother and/or father [28–30].

#### Summary measures

For each of the measures listed above, a summary measure representing history of prior exposure was derived using data from waves 1–3 to indicate whether participants reported the outcome (e.g., presence of depressive symptoms, alcohol consumption, good general wellbeing) at no previous waves, at one wave only, or at ≥2 waves.

#### Statistical analyses

All variables had less than 15% missing data, except for the prior ‘poor emotional control’ exposure (37.9% missing), and the prior ‘below average language/literacy’ exposure (16.1% missing). Missing data were handled using multiple imputation. A total of 50 complete data sets were imputed using multiple imputation by chained equations [35]. Logistic regression was used to impute the binary variables, and ordinal logistic regression was used to impute variables with >3 categories, in each case including all other analysis and auxiliary variables (details below) as predictors. To investigate the association between each antecedent (waves 1–3) and concurrent (wave 4) characteristic with self-harm at wave 4, we used separate logistic regression models within a generalised estimating equations framework to account for clustering by school (exchangeable correlation structure within school at wave 4, and robust standard errors). Each model was run twice to obtain estimates of each association (along with confidence interval [CI] and Wald-test p-value) that were unadjusted (univariable model), and then adjusted for sex, age and SEIFA quintile (multivariable model). Finally, sensitivity analyses were conducted to obtain estimates using available full case data (minimal dataset available at: https://figshare.com/articles/dataset/CATS_dataset_PLOSONE_2020_09_30_dta/13174328).

#### Ethical considerations

Ethics approval was granted by the Royal Children’s Hospital Human Research Ethics Committee (#31089). Permission was granted from the Victorian Department of Education and Training and the Catholic Education Office in Melbourne to recruit through their schools.

## Results

The recruited sample (n = 1 239) contained a slightly lower proportion of boys than girls (46% boys; 54% girls) compared with census data for 8–9 year-old schoolchildren across the state of Victoria (51% boys; 49% girls) [36]. Participants scored slightly higher than the Australian population average for SES (mean SEIFA = 1 012, standard deviation [SD] = 67 vs. M = 1 000, SD = 100) [36]. A higher percentage of participants identified as Indigenous compared with all schoolchildren of the same age in Victoria (5% vs. 1%) [36]. At wave 4, 1 067 of the 1 239 recruited students (86.1%) completed the student questionnaire and this is the sample we used for the study (see Table 1).

**Table 1:** Participant baseline demographic characteristics and self-harm reported at wave 4 (age 11–12 years) (multiple imputation analysis; N = 1067).

Child demographic characteristic	n (%)^a^	Prevalence of self-harm^b^ (%; 95% CI)
Sex		
Girls	562 (52.7)	3.2 (2.1 to 5.0)
Boys	505 (47.3)	2.0 (1.1 to 3.5)
SEIFA^**c**^ quintile		
1^st^ quintile (most advantaged)	383 (35.9)	2.3 (1.3 to 4.1)
2^nd^ quintile	312 (29.2)	2.0 (1.0 to 4.2)
3^rd^ quintile	173 (16.2)	3.0 (1.3 to 6.6)
4^th^ quintile	84 (7.9)	5.6 (2.5 to 12.4)
5^th^ quintile (most disadvantaged)	114 (10.7)	3.4 (1.3 to 8.5)
Country of birth		
Australia	943 (88.4)	2.4 (1.6 to 3.5)
Other	124 (11.6)	3.9 (1.7 to 9.1)
Mother’s highest level of education		
Tertiary	406 (38.1)	2.3 (1.2 to 4.4)
Vocational^**d**^	302 (28.3)	3.4 (1.8 to 6.3)
Year 12 or less	360 (33.7)	2.1 (1.0 to 4.4)

^a^ Frequency estimates were calculated using imputed percentage estimates and total number of students that completed the wave 4 student questionnaire (N = 1067).

^b^ Estimated marginal (population-average) adjusted prevalence rate.

^c^ Socio-Economic Index For Areas.

^d^ Career or trade-specific qualification (typically shorter than an undergraduate degree).

Self-harm data were available for 1 059 participants (96.4% of all 1 067) participants at wave 4. Twenty-eight participants (2.6%) reported past-year self-harm, of whom 18 (64.3%) were females and 10 (35.7%) were males. Ten (35.7%) reported self-battering, 8 (28.6%) reported cutting, 2 (7.1%) reported choking/hanging, 2 (7.1%) reported scratching, and one (3.6%) reported jumping from a height. A further five participants (17.9%) did not specify the nature of their self-harm. Table 1 shows descriptive estimates (obtained via multiple imputation) of the characteristics of the whole sample (n = 1 067): 47.5% were males and 52.5% were females, with a mean age of 11.9 years (SD = 0.39, range 10.7 to 13.4 years) at wave 4.

Table 2 displays unadjusted and adjusted estimates of the associations between participants’ demographic, health, social, educational, and family-related factors at wave 4 and self-harm at wave 4 (obtained via multiple imputation). These cross-sectional results indicated that the presence of depressive symptoms, being in mid-puberty or late/post-puberty, and experiencing difficulties with peer relationships were most strongly associated with self-harm at wave 4. Estimates obtained from available case analysis (conducted as a sensitivity analysis) were similar and are displayed in S1 Table.

**Table 2:** Cross-sectional associations between participant characteristics at wave 4 (age 11–12 years) self-harm reported at wave 4 (multiple imputation analysis; N = 1067).

Child characteristic		n^a^	Characteristics of the cohort	Prevalence of self-harm^b^	Unadjusted association	Adjusted association^c^
			% (95% CI)	% (95% CI)	Odds ratio^d^ (95% CI)	Odds ratio^d^ (95% CI)
***Demographics***						
Age	(centred around 12.0)	-	-	-	0.97 (0.38 to 2.44)	0.99 (0.39 to (2.48)
Sex	Girls	562	52.7 (49.7 to 55.7)	3.2 (2.1 to 5.0)	ref	ref
	Boys	505	47.3 (44.3 to 50.3)	2.0 (1.1 to 3.5)	0.59 (0.27 to 1.28)	1.63 (0.28 to 1.33)
***Mental health***^***e***^						
Depressive symptoms	No	925	86.7 (84.7 to 88.8)	1.2 (0.7 to 1.9)	ref	ref
	Yes	142	13.3 (11.2 to 15.3)	12.0 (7.5 to 18.7)	12.72 (6.02 to 26.88)	11.57 (5.39 to 24.83)
Anxiety symptoms	No	991	92.9 (91.3 to 94.5)	2.3 (1.5 to 3.4)	ref	ref
	Yes	76	7.1 (5.5 to 8.7)	6.3 (2.5 to 15.0)	3.33 (1.20 to 9.24)	2.90 (1.01 to 8.35)
Poor emotional control	No	957	89.7 (87.9 to 91.5)	2.0 (1.4 to 2.9)	ref	ref
	Yes	110	10.3 (8.5 to 12.1)	8.0 (4.2 to 14.9)	3.94 (1.72 to 8.98)	4.22 (1.86 to 9.57)
Good general wellbeing	No	863	80.9 (78.5 to 83.2)	3.2 (2.3 to 4.4)	ref	ref
	Yes	204	19.1 (16.8 to 21.5)	0.5 (0.1 to 3.0)	0.14 (0.02 to 0.99)	0.15 (0.02 to 1.03)
***Peer relationships***^**e**^						
Quantity of friends	Lots	771	72.3 (69.6 to 74.9)	2.0 (1.3 to 3.1)	ref	ref
	Some	256	24.0 (21.4 to 26.6)	2.5 (1.2 to 5.1)	1.36 (0.55 to 3.36)	1.26 (0.51 to 3.14)
	Not many	39	3.7 (2.6 to 4.9)	15.0 (6.7 to 30.2)	9.10 (3.35 to 24.66)	8.74 (3.16 to 24.18)
Conflict with peers	Never	317	29.7 (26.9 to 3.4)	1.5 (0.6 to 3.5)	ref	ref
	<once a month	497	46.6 (43.6 to 49.6)	2.2 (1.2 to 3.9)	1.50 (0.52 to 4.36)	1.48 (0.51 to 4.30)
	≥once a month	253	23.7 (21.2 to 26.3)	4.6 (2.6 to 7.9)	3.23 (1.13 to 9.26)	3.15 (1.10 to 9.04)
Teased frequently	No	920	86.2 (84.2 to 88.3)	1.7 (1.1 to 2.6)	ref	ref
	Yes	147	13.8 (11.7 to 15.8)	8.8 (5.2 to 14.7)	5.04 (2.39 to 10.64)	5.56 (2.62 to 11.83)
Left out frequently	No	981	91.9 (90.3 to 93.6)	2.1 (1.5 to 3.1)	ref	ref
	Yes	86	8.1 (6.4 to 9.7)	7.9 (3.8 to 15.7)	4.01 (1.68 to 9.57)	3.91 (1.62 to 9.44)
Physically hurt frequently	No	1032	96.7 (95.6 to 97.8)	2.2 (1.5 to 3.2)	ref	ref
	Yes	35	3.3 (2.2 to 4.4)	14.2 (6.0 to 30.1)	6.78 (2.44 to 18.83)	7.31 (2.57 to 20.76)
Talked about frequently	No	928	87.0 (84.9 to 89.0)	1.8 (1.2 to 2.6)	ref	ref
	Yes	139	13.0 (11.0 to 15.1)	9.3 (5.4 to 15.5)	6.01 (2.90 to 12.47)	5.70 (2.70 to 12.03)
Victimised online frequently	No	1049	98.3 (97.5 to 99.1)	2.4 (1.6 to 3.4)	ref	ref
	Yes	18	1.7 (0.9 to 2.5)	16.2 (5.1 to 41.0)	8.82 (2.41 to 32.28)	7.98 (2.10 to 30.26)
Bullied (any type) frequently	No	799	74.9 (72.3 to 77.6)	1.3 (0.8 to 2.1)	ref	ref
	Yes	268	25.1 (22.4 to 27.7)	7.5 (5.0 to 11.3)	6.44 (3.12 to 13.27)	6.35 (3.05 to 13.20)
***Puberty (Pubertal stage)***^***e***^	Pre-/early-puberty	388	36.4 (33.5 to 39.3)	0.5 (0.1 to 1.9)	ref	ref
	Mid-puberty	508	47.6 (44.5 to 50.6)	3.1 (2.0 to 5.0)	5.84 (1.39 to 24.58)	6.46 (1.49 to 28.10)
	Late-/post puberty	171	16.0 (13.8 to 18.2)	6.7 (3.4 to 12.8)	11.41 (2.64 to 49.41)	14.36 (2.93 to 70.42)
***Academic performance***						
Teacher report–numeracy^g^	Average or above	872	81.7 (79.3 to 84.1)	2.6 (1.8 to 3.8)	ref	ref
	Below average	195	18.3 (15.9 to 20.7)	2.4 (1.0 to 5.5)	0.94 (0.36 to 2.47)	0.90 (0.34 to 2.39)
Teacher report–literacy^g^	Average or above	870	81.5 (79.1 to 83.9)	2.6 (1.8 to 3.8)	ref	ref
	Below average	197	18.5 (16.1 to 20.9)	2.6 (1.1 to 5.9)	0.90 (0.34 to 2.39)	0.99 (0.37 to 2.67)
Skipped class^e^	No	1022	95.8 (94.6 to 97.0)	2.3 (1.7 to 3.4)	ref	ref
	Yes	45	4.2 (3.0 to 5.4)	7.5 (2.4 to 21.1)	3.06 (0.89 to 10.53)	3.41 (0.96 to 12.09)
***Alcohol consumption***^**e**^						
Had more than a sip	No	918	86.0 (83.9 to 88.1)	1.7 (1.0 to 2.7)	ref	ref
	Yes	149	14.0 (11.9 to 16.1)	8.1 (4.5 to 14.1)	4.07 (1.87 to 8.86)	5.15 (2.27 to 11.70)
***Anti-social behaviour***^***e***^						
Carried a weapon	No	914	85.7 (83.6 to 87.8)	1.5 (0.9 to 2.4)	ref	ref
	Yes	153	14.3 (12.2 to 16.4)	9.4 (5.5 to 15.6)	5.28 (2.51 to 11.09)	6.87 (3.12 to 15.14)
Stole something worth >$5	No	966	90.5 (88.8 to 92.3)	2.2 (1.5 to 3.2)	ref	ref
	Yes	101	9.5 (7.7 to 11.2)	6.4 (2.9 to 13.6)	2.75 (1.09 to 6.96)	3.09 (1.20 to 7.92)
Beat up somebody	No	1030	96.5 (95.4 to 97.6)	2.6 (1.8 to 3.6)	ref	ref
	Yes	37	3.5 (2.4 to 4.6)	2.9 (0.4 to 18.5)	1.04 (0.14 to 7.95)	1.12 (0.14 to 9.05)
***Family relationships***^**e**^						
Discuss feelings with mother	No	744	69.7 (66.9 to 72.4)	2.1 (1.3 to 3.3)	ref	ref
	Yes	323	30.3 (27.6 to 33.1)	3.8 (2.2 to 6.5)	1.72 (0.81 to 6.65)	1.84 (0.85 to 3.97)
Discuss feelings with father	No	453	42.5 (39.5 to 45.5)	2.0 (1.1 to 3.6)	ref	ref
	Yes	614	57.5 (54.5 to 60.5)	3.1 (2.0 to 4.7)	1.71 (0.78 to 3.75)	1.56 (0.70 to 3.44)
Participant has siblings	No	62	5.8 (4.4 to 7.2)	1.4 (0.2 to 9.3)	ref	ref
	Yes	1005	94.2 (92.8 to 95.6)	2.7 (1.9 to 3.7)	1.77 (0.24 to 13.20)	1.91 (0.25 to 14.38)

^a^ Frequency estimates were calculated using imputed percentage estimates and total number of students that completed the wave 4 student questionnaire (N = 1067).

^b^ Estimated marginal (population-average) adjusted prevalence rate.

^c^ Adjusted for age (in years, centred around 12.0 years), sex and SEIFA advantage/disadvantage quintile (estimate for age adjusted for sex and SEIFA, and estimate for sex adjusted for age and SEIFA).

^d^ Odds ratio (OR) comparing odds of child having self-harmed relative to the reference category.

^e^ Child self-report.

^f^ Frequently (at least once per week) teased, left out on purpose, physically hurt, talked about behind back, or victimised online.

^g^ Teacher-report.

Table 3 displays the estimated associations between health, social, academic and family characteristics in waves 1–3 and self-harm at wave 4 (obtained via multiple imputation). A similar pattern of variables that were concurrent correlates of self-harm at wave 4 were also antecedents of self-harm. Importantly, the presence of these characteristics at two or more waves was typically associated with larger adjusted odds ratios than the presence at one wave only. Estimates obtained from available cases analysis (conducted as a sensitivity analysis) were similar and are displayed in S2 Table.

**Table 3:** Associations between wave 1–3 (age 8–9 years to 10–11 years) health, social, academic and family characteristics and self-harm reported at wave 4 (age 11–12 years) (multiple imputation analysis; N = 1067).

Child characteristic	n^a^	Characteristics of the cohort	Prevalence of self-harm^b^	Unadjusted association	Adjusted Association^c^
		% (95% CI)	% (95% CI)	Odds ratio^d^ (95% CI)	Odds ratio^d^ (95% CI)
***Mental Health***					
Depressive symptoms^e^					
No waves	630	59.0 (55.8 to 62.1)	1.0 (0.4 to 2.2)	ref	ref
One wave	290	27.2 (24.3 to 30.0)	3.7 (2.0 to 6.8)	4.10 (1.38 to 12.18)	3.96 (1.33 to 11.76)
Two or three waves	147	13.8 (11.6 to 16.0)	7.1 (3.8 to 13.0)	8.25 (2.76 to 24.69)	7.84 (2.59 to 23.73)
Anxiety symptoms^e^					
No waves	762	71.4 (68.5 to 74.3)	1.3 (0.7 to 2.5)	ref	ref
One wave	214	20.1 (17.5 to 22.6)	6.4 (3.6 to 10.9)	4.96 (2.07 to 11.93)	5.13 (2.10 to 12.54)
Two or three waves	91	8.5 (6.8 to 10.3)	3.8 (1.3 to 11.1)	3.32 (0.89 to 12.47)	3.03 (0.80 to 11.38)
Poor emotional control^f^					
No waves	640	60.0 (56.4 to 63.6)	2.0 (1.1 to 3.6)	ref	ref
One wave	191	17.9 (15.1 to 20.7)	4.2 (2.0 to 8.7)	2.08 (0.75 to 5.73)	2.19 (0.78 to 6.15)
Two or three waves	236	22.1 (19.1 to 25.1)	2.8 (1.0 to 7.4)	1.34 (0.35 to 5.12)	1.42 (0.37 to 5.47)
Good general wellbeing					
No waves	659	61.8 (58.8 to 64.9)	3.6 (2.4 to 5.3)	ref	ref
One wave	256	24.0 (21.3 to 26.8)	1.0 (0.3 to 3.7)	0.27 (0.07 to 1.13)	0.28 (0.07 to 1.14)
Two or three waves	150	14.1 (11.9 to 16.3)	0.8 (0.1 to 5.0)	0.22 (0.03 to 1.55)	0.22 (0.03 to 1.51)
***Peer relationships***^e^					
Not many friends					
No waves	921	86.3 (84.1 to 88.4)	1.6 (1.0 to 2.6)	ref	ref
One wave	98	9.2 (7.4 to 11.1)	7.4 (3.5 to 15.2)	4.56 (1.73 to 12.02)	4.89 (1.83 to 13.08)
Two or three waves	48	4.5 (3.2 to 5.8)	10.6 (4.2 to 25.5)	7.61 (2.41 to 24.05)	7.43 (2.31 to 23.92)
Conflict with peers (≥ once per month)					
No waves	448	42.0 (38.9 to 45.0)	1.3 (0.6 to 2.9)	ref	ref
One wave	410	38.4 (35.3 to 41.4)	3.3 (2.0 to 5.6)	2.59 (0.95 to 7.04)	2.59 (0.95 to 7.06)
Two or three waves	209	19.6 (17.2 to 22.1)	3.5 (1.7 to 7.2)	2.82 (0.90 to 8.82)	2.77 (0.89 to 8.66)
Teased frequently					
No waves	627	58.8 (55.7 to 61.9)	0.6 (0.3 to 1.6)	ref	ref
One wave	273	25.6 (22.8 to 28.4)	3.8 (2.1 to 6.9)	5.88 (1.77 to 19.52)	6.26 (1.91 to 20.55)
Two or three waves	166	15.6 (13.3 to 17.8)	8.8 (5.2 to 14.3)	14.95 (4.87 to 45.95)	15.14 (4.95 to 46.28)
Left out frequently					
No waves	869	81.4 (78.9 to 83.8)	1.5 (1.0 to 2.5)	ref	ref
One wave	171	16.0 (13.7 to 18.3)	7.4 (4.2 to 12.6)	5.07 (2.28 to 11.28)	5.09 (2.28 to 11.37)
Two waves	28	2.6 (1.6 to 3.6)	7.1 (1.8 to 24.8)	5.53 (1.21 to 25.32)	4.89 (1.04 to 23.04)
Physically hurt frequently					
No waves	793	74.3 (71.5 to 77.0)	1.0 (0.5 to 2.0)	ref	ref
One wave	194	18.2 (15.7 to 20.7)	6.5 (3.6 to 11.3)	5.75 (2.27 to 14.53)	6.91 (2.67 to 17.89)
Two or three waves	81	7.6 (5.8 to 9.3)	8.6 (3.9 to 17.7)	8.03 (2.79 to 23.10)	9.42 (3.20 to 27.71)
Talked about frequently					
No waves	775	72.6 (69.8 to 75.4)	1.4 (0.8 to 2.5)	ref	ref
One wave	224	21.0 (18.4 to 23.5)	4.5 (2.4 to 8.3)	3.26 (1.33 to 7.96)	3.25 (1.33 to 7.93)
Two waves	69	6.5 (4.9 to 8.1)	9.7 (4.4 to 20.1)	8.25 (3.05 to 22.30)	7.47 (2.70 to 20.66)
Bullied (any type) frequently^g^					
No waves	456	42.7 (39.5 to 45.8)	0.3 (0.1 to 1.7)	ref	ref
One wave	317	29.7 (26.8 to 32.6)	2.0 (0.9 to 4.6)	6.00 (0.81 to 44.36)	6.78 (0.94 to 49.07)
Two waves	294	27.6 (24.8 to 30.5)	6.9 (4.5 to 10.6)	23.05 (3.53 to 150.55)	24.63 (3.83 to 158.21)
***Academic performance***^***h***^					
Numeracy–below average					
No waves	713	66.8 (63.8 to 69.8)	2.2 (1.4 to 3.6)	ref	ref
One wave	148	13.9 (11.7 to 16.2)	2.6 (0.9 to 7.0)	1.33 (0.41 to 4.36)	1.15 (0.35 to 3.84)
Two or three waves	206	19.3 (16.8 to 21.8)	3.7 (1.8 to 7.7)	1.85 (0.72 to 4.77)	1.70 (0.64 to 4.47)
Literacy–below average					
No waves	717	67.2 (64.2 to 70.2)	2.4 (1.5 to 3.9)	ref	ref
One wave	110	10.3 (8.3 to 12.3)	1.5 (0.2 to 8.8)	0.62 (0.09 to 4.48)	0.60 (0.08 to 4.41)
Two or three waves	240	22.5 (19.8 to 25.2)	3.5 (1.7 to 7.0)	1.37 (0.56 to 3.34)	1.46 (0.58 to 3.68)
***Alcohol consumption (wave 3 only)***^e^					
Had more than a sip of alcohol					
No	905	84.8 (82.5 to 87.0)	2.0 (1.3 to 3.1)	ref	ref
Yes	162	15.2 (13.0 to 17.5)	5.7 (2.9 to 10.8)	2.46 (1.05 to 5.77)	2.95 (1.23 to 7.06)
***Family relationships***^e^					
Does not discuss feelings with mother					
No waves	538	50.4 (47.3 to 53.5)	1.9 (1.0 to 3.5)	ref	ref
One wave	245	23.0 (20.3 to 25.7)	3.6 (1.9 to 6.9)	1.87 (0.73 to 4.82)	1.93 (0.74 to 5.06)
Two or three waves	284	26.6 (23.8 to 29.4)	3.0 (1.5 to 5.9)	1.43 (0.54 to 3.75)	1.58 (0.58 to 4.31)
Does not discuss feelings with father					
No waves	230	28.1 (25.3 to 30.9)	1.5 (0.6 to 4.0)	ref	ref
One wave	245	23.0 (20.4 to 25.7)	3.0 (1.5 to 6.2)	2.11 (0.57 to 7.84)	2.04 (0.55 to 7.54)
Two or three waves	522	48.9 (45.8 to 52.0)	2.9 (1.7 to 4.8)	2.06 (0.65 to 6.55)	1.95 (0.61 to 6.17)

^a^ Frequency estimates were calculated using imputed percentage estimates and total number of students that completed the wave 4 student questionnaire (N = 1067).

^b^ Estimated marginal (population-average) adjusted prevalence rate.

^c^ Adjusted for age (in years, centred around 12.0 years), sex, and SEIFA advantage/disadvantage quintile.

^d^ Odds ratio (OR) comparing odds of child having self-harmed relative to the reference category.

^e^ Child self-report.

^f^ Parent-report.

^g^ Student classified as frequently bullied if experienced any of the following at least once per week: teased, left out on purpose, physically hurt, talked about behind back, or victimised online.

^h^ Teacher-report.

## Discussion

In this prospective cohort study examining the prevalence, correlates and antecedents of self-harm in a population-based sample of children in Melbourne, Australia, we found that 3% of 11-12-year-olds had self-harmed during the previous 12 months. To our knowledge, these are the first prevalence estimates of self-harm among a community-dwelling sample of primary school-aged children internationally. Our findings suggest that mental health, puberty and peer relationships are most strongly associated with self-harm among primary school-aged children. Participants who had few friends, and those who had experienced bullying victimisation, were seven and 24 times more likely to have self-harmed at age 11–12 years, respectively. Additionally, participants were experienced frequent social exclusion by their peers, and those who had been teased frequently, were four and 15 times more likely to have self-harmed, respectively. These findings add weight to the predictive impact of bullying victimisation on self-harm in young people [37, 38] and demonstrate that this impact is observable at a considerably younger age than has previously been reported. In a recent prospective multicentre study from Europe examining life events as risk factors for self-harm in adolescents aged 14–15 years, Kaess et al. [38] demonstrated that prior bullying victimisation was associated with the first onset of self-harm at 12 month follow-up. Our finding also supports the conclusion of a recent meta-analysis which demonstrated an association between bullying and cyberbullying victimisation and self-harm in young people [39]. In terms of mental health, participants who self-harmed were more than seven times more likely to experience depressive symptoms and five times more likely to experience anxiety than their peers who had not self-harmed.

Life-course theorists have long posited that the period between childhood and adolescence is a critical developmental period during which social learning and interaction with peers become central developmental functions [40]. Young people begin to enact various social and behavioural strategies during this period and receive prompt feedback about the success (or lack thereof) of the chosen strategies [41]. As such, experiences during this period can have broad-ranging implications for children’s later social development and can be expected to affect many areas of behaviour, from attachment to aggression and sexuality. For participants in our study who experienced persistent difficulties with their peer groups and later self-harmed, it is possible that this self-harm may contribute to further social isolation due to the stigma associated with such behaviour [42]. Other potential mechanisms may also contribute to self-harm in children and adolescence. These include the influence of sex hormones, which act on hippocampal and hypothalamic systems and shift control of affect and cognitive process, may contribute to the onset and rate of self-harm [11]. Potential mechanisms also include factors related to cognitive development, as evidence suggests that many young people who self-harm demonstrate specific deficits in problem-solving abilities [43]. Similarly, difficulties with emotion regulation may precipitate self-harm in young people; participants who reported poor emotional control in our sample were more than four times as likely to have self-harmed. This finding supported those of a recent study by Palmer et al. examining the association between emotion dysregulation and the onset of self-harm in adolescents aged 14–15 years in the UK [44]. Palmer et al. reported that, prior to the onset of first self-harm, participants experienced difficulties regulating their emotions and, specifically, a lack of emotional clarity and poor impulse control. These difficulties in regulating emotions, the authors argued, might help to identify adolescents at increased risk of engaging in self-harm in the future.

In our study we did not assess the presence or degree of suicidal intent when measuring self-harm and were therefore unable to make inferences about the prevalence, antecedents or characteristics associated with non-suicidal self-injury (NSSI). We adopted this approach, in part, due to the many limitations of NSSI as a concept [45]; first, the definition of NSSI is restricted to methods such as cutting, burning, stabbing, and hitting, thus ensuring that any act of non-suicidal self-poisoning cannot be classified as NSSI [45]. Second, longitudinal research has identified NSSI as one of the most important risk factors for suicide attempts [46], indicating that intentionality can change over time. Third, as many people use different methods of self-harm on different occasions [47], it is possible that people may engage in both NSSI and self-poisoning at different times, which would result in an under-ascertainment of self-harming behaviours if the NSSI categorisation alone was used.

## Strengths and limitations

Strengths of our study include its prospective design, multiple assessment points across a narrow age range, large population-based sample size, comprehensive measures of emotional and behavioural problems, and inclusion of both males and females. Additionally, we scrutinised all participants’ free-text descriptions of self-reported self-harm events in order to improve case ascertainment. Unlike many previous studies [13, 16, 18], our sample was a non-treatment-seeking sample and participants did not need to have sought medical help for self-harm to have been included. Our study also contained some potential limitations. First, the number of participants who had self-harmed by age 11–12 years was relatively small; however, these participants formed part of a much larger, population-based community cohort for whom we have comprehensive demographic, mental health, social, family composition, and educational data. Second, an active parental consent process was employed at recruitment, with only 54% of parents providing written consent. Third, the sample was skewed towards higher SES and had a higher proportion of Indigenous children than the general Australian population. Fourth, we did not ask about self-harm prior to wave 4 and this may represent a missed opportunity to collect informative data. Fifth, self-harm was measured via self-report and this may have contributed to an under-ascertainment of cases [48]. Finally, we did not assess the presence or degree of suicidal intent when measuring self-harm and were therefore unable to make inferences about the prevalence, antecedents or characteristics associated with NSSI. As discussed above, however, this approach resulted in a larger proportion of all self-harming behaviours being detected.

## Conclusion

Primary school-aged children who experience persistent difficulties within their peer group, including bullying, social exclusion and teasing, are at increased risk of self-harm as they progress through the pubertal transition. These peer problems, along with the early onset of puberty, dominate the risk profile for self-harm among children at this age and represent tangible, modifiable risk factors which may benefit from targeted prevention initiatives. The focus of existing intervention efforts aimed at preventing and reducing adolescent self-harm should extend to primary school-aged children, with a particular focus on mental health and peer relationships during the pubertal transition.

## Supporting information

S1 Fig “Participant recruitment and retention across waves 1–4 of the Childhood to Adolescence Transition Study (CATS)”. (DOCX)

S1 File Additional information about the recruitment procedure and measurement of depressive symptoms, anxiety symptoms, emotional control, and overall wellbeing. (DOCX)

S1 Table “Cross-sectional associations between participant characteristics at wave 4 (age 11–12 years) and self-harm reported at wave 4 (available case analysis)”. (DOCX)

S2 Table “Associations between wave 1–3 (age 8–9 years to 10–11 years) health, social, academic and family characteristics and self-harm reported at wave 4 (age 11–12 years) (available case analysis)”. (DOCX)
