# Supplementary material for: Self-harm in primary school-aged children: Prospective cohort study
Source: PLoS One. 2020 Nov 30;15(11):e0242802. doi: 10.1371/journal.pone.0242802 (PMC7703962; doi:10.1371/journal.pone.0242802)

**S1 Fig.** **Participant recruitment and retention across waves 1-4 of the Childhood to Adolescence Transition Study (CATS).**


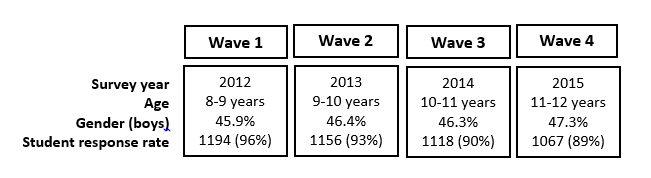

Supplement: S1 Fig — (DOCX) [file pone.0242802.s001.docx]
