# Supplementary material for: Self-harm in primary school-aged children: Prospective cohort study
Source: PLoS One. 2020 Nov 30;15(11):e0242802. doi: 10.1371/journal.pone.0242802 (PMC7703962; doi:10.1371/journal.pone.0242802)
Supplement: S1 File — (DOCX) [file pone.0242802.s002.docx]

**Methodology**

**Procedure**

Research assistants visited each school annually and participants completed questionnaires using electronic tablets. At waves 1 and 2 the questionnaire took approximately 20-30 minutes to complete. At waves 3 and 4, the questionnaire was slightly longer (approximately 40-50 minutes). Participants who changed schools were followed and completed the assessment at school, home, a community centre, or The Royal Children’s Hospital in Melbourne.

***Depressive symptoms***

At waves 1-3, depressive symptoms were measured using two items from the SMFQ (‘I felt miserable and unhappy’ and ‘I didn’t enjoy anything at all’), which have been shown to have validity as markers of depressive symptoms in similar age population-based samples (1, 2) Items were scored on a 5-point Likert scale ranging from 0 (never) to 4 (almost always), to match other measures included in the questionnaire. Items were then recoded to a 3-point scale to match the original SMFQ scoring. The sum of the recoded items (ranging from 0-4) was dichotomised to generate a binary variable using the cut-point identified by Rhew and colleagues (2) of no depressive symptoms (≤1) versus depressive symptoms (>1). At wave 4, the full 13-item SMFQ was included. The sum of the 13 items was calculated to generate a total score (ranging from 0-26), which was then dichotomised using a cut-point of >6 to indicate the presence of depressive symptoms (3).

***Anxiety symptoms***

At waves 1-3, anxiety symptoms were measured using two items selected from the SCAS (‘I worry about things’ and ‘I feel afraid’). These items were scored on a 5-point scale (ranging from 0-4 and were then recoded onto a 4-point scale to match the original SCAS scoring. The sum of the two recoded items (ranging from 0-6) was then dichotomised into a binary variable reflecting no anxiety symptoms (≤2) versus anxiety symptoms (>2). The full SCAS was included in the wave 4 student questionnaire. The sum of the 38 items (ranging from 0-114) was rescaled to a standardised T-score (mean = 50; SD = 10). A T-score of >60 reflected the presence of anxiety symptoms (4).

***Emotional control***

The mean score of these four items was calculated, then dichotomised into a binary variable reflecting good emotional control (≥ 2.5) versus poor emotional control (< 2.5). These items were completed by a parent in waves 1-3, and by participant self-report in wave 4.

***Overall wellbeing***

Three items were included at waves 1-2 and six items at waves 3-4.

References

1. Angold A, Costello EJ, Messer SC, Pickles A. Development of a short questionnaire for use in epidemiological studies of depression in children and adolescents. International journal of methods in psychiatric research. 1995.

2. Rhew IC, Simpson K, Tracy M, Lymp J, McCauley E, Tsuang D, et al. Criterion validity of the Short Mood and Feelings Questionnaire and one-and two-item depression screens in young adolescents. Child and adolescent psychiatry and mental health. 2010;4(1):8.

3. Katon W, Russo J, Richardson L, McCauley E, Lozano P. Anxiety and depression screening for youth in a primary care population. Ambulatory Pediatrics. 2008;8(3):182-8.

4. Nauta MH, Scholing A, Rapee RM, Abbott M, Spence SH, Waters A. A parent-report measure of children’s anxiety: psychometric properties and comparison with child-report in a clinic and normal sample. Behaviour research and therapy. 2004;42(7):813-39.
