# Supplementary material for: Self-harm in primary school-aged children: Prospective cohort study
Source: PLoS One. 2020 Nov 30;15(11):e0242802. doi: 10.1371/journal.pone.0242802 (PMC7703962; doi:10.1371/journal.pone.0242802)
Supplement: S1 Table — (DOCX) [file pone.0242802.s003.docx]

| **S1 Table. Cross-sectional associations between participant characteristics at wave 4 (age 11-12 years) and self-harm reported at wave 4 (available case analysis).** | | | | |
| --- | --- | --- | --- | --- |
| **Participant characteristic** | **n (%)^a^** | **Prevalence of self-harm^b^** | **Unadjusted association** | **Adjusted**  **association^c^** |
|  |  |  | Odds ratio^d^ (95% CI) | Odds ratio^d^ (95% CI) |
| *Overall sample* | 1059 (100.0) | 28 (2.6) |  |  |
| *Demographics* |  |  |  |  |
| Age (years, centred around 12 years) | - | - | 0.96 (0.38 to 2.44) | 0.99 (0.38 to 2.56) |
| Gender |  |  |  |  |
| Girls | 556 (52.5) | 18 (3.2) | ref | ref |
| Boys | 503 (47.5) | 10 (2.0) | 0.60 (0.28 to 1.32) | 0.57 (0.25 to 1.27) |
| SEIFA disadvantage/advantage quintile |  |  |  |  |
| 1^st^ quintile (most advantaged) | 380 (36.3) | 8 (2.1) | ref | ref |
| 2^nd^ quintile | 303 (28.9) | 5 (1.7) | 0.71 (0.25 to2.05) | 0.71 (0.24 to 2.06) |
| 3^rd^ quintile | 170 (16.2) | 5 (2.9) | 1.31 (0.45 to 3.78) | 1.35 (0.46 to 3.94) |
| 4^th^ quintile | 84 (8.0) | 5 (6.0) | 2.80 (0.97 to 8.05) | 2.72 (0.93 to 7.92) |
| 5^th^ quintile (least disadvantaged) | 110 (10.5) | 4 (3.6) | 1.60 (0.51 to 5.06) | 1.55 (0.48 to 4.98) |
| *Mental Health^e^* |  |  |  |  |
| Depressive symptoms |  |  |  |  |
| No | 913 (87.4) | 11 (1.2) | ref | ref |
| Yes | 132 (12.6) | 16 (12.1) | 11.66 (5.44 to 24.98) | 11.60 (5.17 to 26.02) |
| Anxiety symptoms |  |  |  |  |
| No | 943 (93.0) | 19 (2.0) | ref | ref |
| Yes | 71 (7.0) | 5 (7.0) | 3.68 (1.33 to 10.17) | 3.14 (1.09 to 9.01) |
| Poor emotional control |  |  |  |  |
| No | 952 (89.9) | 20 (2.1) | ref | ref |
| Yes | 107 (10.1) | 8 (7.5) | 3.85 (1.68 to 8.82) | 4.32 (1.86 to 10.01) |
| Good general wellbeing |  |  |  |  |
| No | 855 (80.7) | 27 (3.2) | ref | ref |
| Yes | 204 (19.3) | 1 (0.5) | 0.14 (0.02 to 1.02) | N/A |
| *Peer relationships^e^* |  |  |  |  |
| Quantity of friends |  |  |  |  |
| Lots | 766 (72.3) | 15 (2.0) | ref | ref |
| Some | 255 (24.1) | 7 (2.8) | 1.42 (.57 to 3.52) | 1.40 (0.55 to 3.56) |
| Not many | 38 (3.6) | 6 (15.8) | 9.44 (3.49 to 25.57) | 9.94 (3.52 to 28.07) |
| Argue/fall out with friends |  |  |  |  |
| Never | 315 (29.8) | 5 (1.6) | ref | ref |
| Less than once per month | 492 (46.6) | 11 (2.2) | 1.42 (0.49 to 4.14) | 1.78 (0.56 to 5.70) |
| At least once per month | 250 (23.7) | 12 (4.8) | 3.17 (1.10 to 9.09) | 3.91 (1.24 to 12.34) |
| Teased frequently |  |  |  |  |
| No | 915 (86.4) | 16 (1.8) | ref | ref |
| Yes | 144 (13.6) | 12 (8.3) | 5.09 (2.42 to 10.71) | 6.29 (2.89 to 13.71) |
| Left out frequently |  |  |  |  |
| No | 972 (92.0) | 21 (2.2) | ref | ref |
| Yes | 85 (8.0) | 7 (8.2) | 4.15 (1.73 to 9.95) | 4.38 (1.79 to 10.71) |
| Physically hurt frequently |  |  |  |  |
| No | 1022 (96.8) | 23 (2.3) | ref | ref |
| Yes | 34 (3.2) | 5 (14.7) | 7.47 (2.69 to 20.74) | 8.73 (3.01 to 25.27) |
| Talked about frequently |  |  |  |  |
| No | 921 (87.2) | 15 (1.6) | ref | ref |
| Yes | 135 (12.8) | 13 (9.6) | 5.91 (2.84 to 12.28) | 6.33 (2.93 to 13.70) |
| Victimised online frequently |  |  |  |  |
| No | 1039 (98.4) | 25 (2.4) | ref | ref |
| Yes | 17 (1.6) | 3 (17.7) | 8.83 (2.43 to 32.16) | 8.34 (2.18 to 32.85) |
| Bullied (any type)^f^ frequently |  |  |  |  |
| No | 793 (75.2) | 9 (1.1) | ref | ref |
| Yes | 261 (24.8) | 19 (7.3) | 6.37 (3.08 to 13.17) | 7.42 (3.38 to 16.27) |
| *Puberty^e^* |  |  |  |  |
| Pubertal stage |  |  |  |  |
| Pre puberty/Early puberty | 382 (36.8) | 2 (0.5) | ref | ref |
| Mid puberty | 494 (47.6) | 16 (3.2) | 6.26 (1.49 to 26.33) | 6.56 (1.49 to 28.86) |
| Late puberty/Post puberty | 162 (15.6) | 10 (6.2) | 12.38 (2.83 to 54.11) | 12.58 (2.46 to 64.39) |
| *Academic performance* |  |  |  |  |
| Teacher report – numeracy^g^ |  |  |  |  |
| Average or above | 836 (82.0) | 23 (2.8) | ref | ref |
| Below average | 184 (18.0) | 5 (2.7) | 1.02 (0.38 to 2.70) | 0.77 (0.26 to 2.27) |
| Teacher report – literacy^g^ |  |  |  |  |
| Average or above | 839 (82.0) | 23 (2.7) | ref | ref |
| Below average | 184 (18.0) | 5 (2.7) | 0.99 (0.37 to 2.64) | 0.88 (0.29 to 2.63) |
| Skipped class*^e^* |  |  |  |  |
| No | 1016 (95.9) | 25 (2.5) | ref | ref |
| Yes | 43 (4.1) | 3 (7.0) | 3.00 (0.88 to 10.31) | 3.91 (1.09 to 14.09) |
| *Alcohol consumption (past 12 months)^e^* |  |  |  |  |
| Had more than a sip of alcohol |  |  |  |  |
| No | 903 (86.2) | 17 (1.9) | ref | ref |
| Yes | 145 (13.8) | 11 (7.6) | 4.26 (1.96 to 9.29) | 6.19 (2.66 to 14.39) |
| *Anti-social behaviour^e^* |  |  |  |  |
| Carried a weapon |  |  |  |  |
| No | 907 (85.7) | 15 (1.7) | ref | ref |
| Yes | 152 (14.4) | 13 (8.6) | 5.69 2.71 to 11.96 | 7.05 (3.11 to 15.96) |
| Stolen something worth more than $5 |  |  |  |  |
| No | 961 (90.8) | 22 (2.3) | ref | ref |
| Yes | 98 (9.3) | 6 (6.1) | 2.74 1.08 to 6.93 | 3.37 (1.29 to 8.80) |
| Beat up somebody |  |  |  |  |
| No | 1019 (96.7) | 27 (2.7) | ref | ref |
| Yes | 35 (3.3) | 1 (2.9) | 1.08 (0.14 to 8.18) | 1.34 (0.17 to 10.78) |
| *Family relationships^e^* |  |  |  |  |
| Discuss feelings with mother |  |  |  |  |
| Yes | 734 (69.6) | 16 (2.2) | ref | ref |
| No | 320 (30.4) | 12 (3.8) | 1.75 (0.82 to 3.71) | 2.03 (0.93 to 4.45) |
| Discuss feelings with father |  |  |  |  |
| Yes | 443 (43.0) | 9 (2.0) | ref | ref |
| No | 587 (57.0) | 18 (3.1) | 1.57 (0.70 to 3.50) | 1.58 (0.67 to 3.68) |
| Child have siblings |  |  |  |  |
| No | 62 (5.9) | 1 (1.6) | ref | ref |
| Yes | 997 (94.2) | 27 (2.7) | 1.69 (0.23 to 12.61) | 1.81 (0.24 to 13.89) |

^a^ Percentage out of sample with observed data of corresponding variable

^b^ Percentage out of row total

^c^ Adjusted for age (in years, centred around 12 years), gender, and SEIFA advantage/disadvantage quintile (estimate for age adjusted for sex, and SEIFA and estimate for sex adjusted for age and SEIFA).

^d^ Odds ratio (OR) comparing odds of child having self-harmed in category relative to the reference

^e^ Child self-report

^f^ Student classified as frequently bullied if experienced any of the following at least once per week: teased, left out on purpose, physically hurt, talked about behind back, or victimised online

^g^ Teacher-report
