# Supplementary material for: Self-harm in primary school-aged children: Prospective cohort study
Source: PLoS One. 2020 Nov 30;15(11):e0242802. doi: 10.1371/journal.pone.0242802 (PMC7703962; doi:10.1371/journal.pone.0242802)
Supplement: S2 Table — (DOCX) [file pone.0242802.s004.docx]

**S2 Table. Associations between wave 1-3 (age 8-9 years to 10-11 years) health, social, academic and family characteristics and self-harm reported at wave 4 (age 11-12 years) (available case analysis).**

| **Participant characteristic** | **n (%)^a^** | **Prevalence of self-harm^b^** | **Unadjusted**  **association** | **Adjusted**  **association^c^** |
| --- | --- | --- | --- | --- |
|  |  |  | Odds ratio^d^ (95% CI) | Odds ratio^d^ (95% CI) |
| *Overall sample* | 1059 (100.0) | 28 (2.6) |  | - |
| *Mental Health* |  |  |  |  |
| Depressive symptoms^e^ |  |  |  |  |
| No waves | 554 (60.4) | 5 (0.90) | ref | ref |
| One wave | 246 (26.8) | 10 (4.1) | 4.67 (1.58 to 13.78) | 5.60 (1.72 to 18.26) |
| Two or three waves | 118 (12.9) | 10 (8.5) | 10.21 (3.43 to 30.35) | 11.66 (3.50 to 38.88) |
| Anxiety symptoms^e^ |  |  |  |  |
| No waves | 673 (72.8) | 8 (1.2) | ref | ref |
| One wave | 180 (19.5) | 12 (6.7) | 5.94 (2.39 to 14.78) | 9.93 (2.30 to 15.30) |
| Two or three waves | 72 (7.8) | 3 (4.2) | 3.61 (0.94 to 13.95) | 3.16 (0.81 to 12.37) |
| Poor emotional control^f^ |  |  |  |  |
| No waves | 397 (60.2) | 7 (1.8) | ref | ref |
| One wave | 122 (18.5) | 6 (4.9) | 3.00 (0.99 to 9.14) | 3.23 (1.03 to 10.14) |
| Two or three waves | 140 (21.2) | 3 (2.1) | 1.23 (0.31 to 4.93) | 1.29 (0.32 to 5.17) |
| Good general wellbeing^e^ |  |  |  |  |
| No waves | 576 (61.2) | 20 (3.5) | ref | ref |
| One wave | 230 (24.4) | 2 (0.9) | 0.25 (0.06 to 1.04) | 0.24 (0.06 to 1.03) |
| Two or three waves | 135 (14.4) | 1 (0.7) | 0.22 (0.03 to 1.53) | N/A |
| *Peer relationships^e^* |  |  |  |  |
| Not many friends |  |  |  |  |
| No waves | 835 (87.3) | 13 (1.6) | ref | ref |
| One wave | 84 (8.8) | 6 (7.1) | 4.89 (1.82 to 13.17) | 5.94 (2.12 to 16.64) |
| Two or three waves | 38 (4.0) | 4 (10.5) | 7.47 (2.32 to 24.06) | 7.99 (2.36 to 27.02) |
| Argue/fall out with friends |  |  |  |  |
| No waves | 417 (42.0) | 5 (1.2) | ref | ref |
| One wave | 383 (38.5) | 13 (3.4) | 2.78 (0.99 to 7.84) | 2.62 (0.91 to 7.51) |
| Two waves | 194 (19.5) | 7 (3.6) | 3.02 (0.95 to 9.54) | 3.14 (0.99 to 10.03) |
| Teased frequently |  |  |  |  |
| No waves | 572 (59.9) | 3 (0.5) | ref | ref |
| One wave | 242 (25.3) | 9 (3.7) | 6.82 (1.94 to 24.03) | 12.92 (2.81 to 59.43) |
| Two or three waves | 141 (14.8) | 12 (8.5) | 16.82 (5.04 to 56.08) | 28.39 (6.40 to 125.97) |
| Left out frequently |  |  |  |  |
| No waves | 816 (82.3) | 12 (1.5) | ref | ref |
| One wave | 150 (15.1) | 11 (7.3) | 5.37 (2.40 to 12.00) | 4.77 (2.06 to 11.03) |
| Two waves | 25 (2.5) | 2 (8.0) | 5.69 (1.22 to 26.39) | 5.48 (1.15 to 26.11) |
| Physically hurt frequently |  |  |  |  |
| No waves | 711 (75.0) | 7 (1.0) | ref | ref |
| One wave | 169 (17.8) | 11 (6.5) | 7.02 (2.67 to 18.43) | 8.62 (3.12 to 23.85) |
| Two or three waves | 68 (7.2) | 6 (8.8) | 9.77 (3.18 to 30.01) | 12.77 (3.98 to 40.93) |
| Talked about frequently |  |  |  |  |
| No waves | 727 (73.9) | 10 (1.4) | ref | ref |
| One wave | 200 (20.3) | 9 (4.5) | 3.28 (1.33 to 8.12) | 2.98 (1.16 to 7.63) |
| Two waves | 57 (5.8) | 6 (10.5) | 8.38 (2.99 to 23.48) | 7.95 (2.76 to 22.87) |
| Frequent online victimisation – wave 3 only |  |  |  |  |
| No | 988 (97.9) | 26 (2.6) | ref | ref |
| Yes | 21 (2.1) | 0 (0.0) | N/A | N/A |
| Bullied (any type) frequently^g^ |  |  |  |  |
| No waves | 405 (43.1) | 1 (0.3) | ref | ref |
| One wave | 280 (29.8) | 5 (1.8) | 6.88 (0.90 to 52.30) | 7.17 (0.81 to 63.18) |
| Two or three waves | 254 (27.1) | 18 (7.1) | 28.34 (4.34 to 185.08) | 35.46 (4.81 to 261.67) |
| *Academic performance^h^* |  |  |  |  |
| Numeracy – below average |  |  |  |  |
| No waves | 595 (67.1) | 11 (1.9) | ref | ref |
| One wave | 123 (13.9) | 3 (2.4) | 1.35 (0.37 to 4.86) | 0.74 (0.16 to 3.41) |
| Two or three waves | 169 (19.1) | 7 (4.1) | 2.41 (0.95 to 6.13) | 2.03 (0.77 to 5.34) |
| Literacy – below average |  |  |  |  |
| No waves | 594 (66.8) | 13 (2.2) | ref | ref |
| One wave | 92 (10.4) | 1 (1.1) | 0.49 (0.07 to 3.77) | 0.55 (0.07 to 4.26) |
| Two or three waves | 203 (22.8) | 7 (3.5) | 1.56 (0.62 to 3.93) | 1.99 (0.75 to 5.24) |
| *Alcohol consumption (wave 3 only)^e^* |  |  |  |  |
| Had more than a sip of alcohol (past twelve months) |  |  |  |  |
| No | 869 (85.1) | 19 (2.2) | ref | ref |
| Yes | 152 (14.9) | 8 (5.3) | 2.42 (1.03 to 5.67) | 3.21 (1.33 to 7.73) |
| *Family relationships^e^* |  |  |  |  |
| Does not discuss feelings with mother |  |  |  |  |
| No waves | 489 (51.0) | 9 (1.8) | ref | ref |
| One wave | 219 (22.8) | 8 (3.7) | 2.08 (0.80 to 5.39) | 2.22 (0.84 to 5.90) |
| Two or three waves | 251 (26.2) | 7 (2.8) | 1.51 (0.56 to 4.09) | 1.49 (0.50 to 4.38) |
| Does not discuss feelings with father |  |  |  |  |
| No waves | 269 (27.8) | 3 (1.1) | ref | ref |
| One wave | 224 (23.2) | 7 (3.1) | 2.82 (0.72 to 10.94) | 2.43 (0.60 to 9.80) |
| Two or three waves | 474 (49.0) | 13 (2.7) | 2.50 (0.72 to 8.70) | 2.40 (0.68 to 8.45) |

^a^ Percentage out of sample with observed data of corresponding variable.

^b^ Percentage out of row total.

^c^ Adjusted for age (in years, centred around 9.0 years) and gender.

^d^ Odds ratio (OR) comparing odds of child having self-harmed in category relative to the reference

^e^ Child self-report

^f^ Parent report

^g^ Student classified as frequently bullied if experienced any of the following at least once per week: teased, left out on purpose, physically hurt, talked about behind back, or victimised online

^h^ Teacher report
